# Validity Testing and Cultural Adaptation of the eHealth Literacy Questionnaire (eHLQ) Among People With Chronic Diseases in Taiwan: Mixed Methods Study

**DOI:** 10.2196/32855

**Published:** 2022-01-19

**Authors:** Yu-Chi Chen, Christina Cheng, Richard H Osborne, Lars Kayser, Chieh-Yu Liu, Li-Chun Chang

**Affiliations:** 1 Institute of Clinical Nursing College of Nursing National Yang Ming Chiao Tung University Taipei Taiwan; 2 Centre for Global Health and Equity Swinburne University of Technology Melbourne Australia; 3 Department of Public Health University of Copenhagen Copenhagen Denmark; 4 Department of Health Care Management National Taipei University of Nursing and Health Sciences Taipei Taiwan; 5 School of Nursing Chang Gung University of Science and Technology Tao-Yuan Taiwan

**Keywords:** chronic illness, eHealth literacy questionnaire, eHLQ, validation, cultural adaptation, eHealth

## Abstract

**Background:**

Advancements in digital technologies seek to promote health and access to services. However, people lacking abilities and confidence to use technology are likely to be left behind, leading to health disparities. In providing digital health services, health care providers need to be aware of users’ diverse electronic health (eHealth) literacy to address their particular needs and ensure equitable uptake and use of digital services. To understand such needs, an instrument that captures users’ knowledge, skills, trust, motivation, and experiences in relation to technology is required. The eHealth Literacy Questionnaire (eHLQ) is a multidimensional tool with 7 scales covering diverse dimensions of eHealth literacy. The tool was simultaneously developed in English and Danish using a grounded and validity-driven approach and has been shown to have strong psychometric properties.

**Objective:**

This study aims to translate and culturally adapt the eHLQ for application among Mandarin-speaking people with chronic diseases in Taiwan and then undertake a rigorous set of validity-testing procedures.

**Methods:**

The cross-cultural adaptation of the eHLQ included translation and evaluation of the translations. The measurement properties were assessed using classical test theory and item response theory (IRT) approaches. Content validity, known-group validity, and internal consistency were explored, as well as item characteristic curves (ICCs), item discrimination, and item location/difficulty.

**Results:**

The adapted version was reviewed, and a recommended forward translation was confirmed through consensus. The tool exhibited good content validity. A total of 420 people with 1 or more chronic diseases participated in a validity-testing survey. The eHLQ showed good internal consistency (Cronbach α=.75-.95). For known-group validity, all 7 eHLQ scales showed strong associations with education. Unidimensionality and local independence assumptions were met except for scale 2. IRT analysis showed that all items demonstrated good discrimination (range 0.27-12.15) and a good range of difficulty (range 0.59-1.67) except for 2 items in scale 7.

**Conclusions:**

Using a rigorous process, the eHLQ was translated from English into a culturally appropriate tool for use in the Mandarin language. Validity testing provided evidence of satisfactory-to-strong psychometric properties of the eHLQ. The 7 scales are likely to be useful research tools for evaluating digital health interventions and for informing the development of health technology products and interventions that equitably suit diverse users’ needs.

## Introduction

In societies with a rapid ongoing service transformation of health care to be more digitally supported and expectations of higher community involvement, it is necessary that people be actively supported to participate in their own care, including engagement with electronic health (eHealth) care resources. People need to be able to obtain relevant health information and support from web-based services, use technology for health management, and receive appropriate care from eHealth service systems [1-3]. In this era of eHealth care management, understanding the eHealth literacy (eHL) of service users is important to ensure they can equitably benefit from and take advantage of the digital services and health technologies [4,5]. People with a range of eHL skills are more likely to engage in eHealth resources, leading to improved knowledge, skills, and confidence to actively manage their health condition [6-9]. Conversely, people with low eHL may not be able to understand, access, and use health and care services and health information, leading to suboptimal disease self-management, increased vulnerability, and poor health outcomes [3,6,10].

Digital health care service solutions need to recognize and respond to users’ personal goals, values, and competence in a sociotechnical context. In terms of the solution’s function and interface design, the solution must not only serve the goals of health service providers but also satisfy diverse users' needs across their eHL levels; this will increase the benefits and upscale the benefits of the solution [11-13].

The concept of eHL was introduced in the Web 1.0 era by Norman and Skinner [1,14] in 2006 and was described as “the ability to seek, find, understand, and appraise health information from electronic sources and apply the knowledge gained to addressing or solving a health problem.” Over the past 2 decades, health services have become more complex and interactive with the expectation that users be active in managing their own condition using digital services. As such, new and more comprehensive tools to measure eHL are required [15]. In response to the advances in health technology, Norgaard et al [5] proposed in 2015 the eHealth Literacy Framework (eHLF), which comprises 7 dimensions of eHL.

The eHLF, developed using a grounded validity-driven approach [16] with extensive international consultation with service users, health professionals, researchers, and technology experts, provides a contemporary and comprehensive map of an individual’s technology health literacy. The eHLF covers knowledge and skills, the eHealth system’s attributes, and how an individual interacts with the system. Subsequently, the eHealth Literacy Questionnaire (eHLQ) was developed based on the eHLF in Danish and English. The tool was tested in Denmark in a large sample of people with chronic diseases and the general population. The questionnaire was found to have a wide range of excellent psychometric properties [17,18].

Today, the far-reaching nature of the digital environment with the internet and cloud technology makes services and information borderless, and issues associated with eHL can also have global ramifications. What is the relationship between eHL and health care behaviors? What is the difference in eHL levels between the people of Taiwan or China and those of other countries? To explore these issues, an appropriate and psychometrically sound evaluation tool is required. Although the eHLQ has undergone validity testing in Denmark in both health and community settings and is available in Danish and English, a Chinese version for use in Taiwan is required. Given that Denmark and Taiwan have different health care systems and that items and constructs may be subject to differential cultural and linguistic interpretations, it is important that careful translation and cultural adaption, as well as psychometric testing, be undertaken to inform researchers, clinicians, and health system managers in Taiwan and other Mandarin-speaking areas. The aim of this study was to translate and culturally adapt the eHLQ from English to Chinese and evaluate its cultural and psychometric properties in a group of Mandarin-speaking people with chronic diseases.

## Methods

### Study Design

This was a 2-phase study. Phase 1 involved the translation and cultural adaptation of the eHLQ for application in the Mandarin language. In phase 2, the Chinese version was psychometrically tested among people with chronic diseases using classical test theory and item response theory (IRT) approaches. IRT, also known as latent response theory, refers to a family of mathematical models that seek to explain the relationship between latent traits (unobservable characteristic or attribute) and their manifestations.

### eHealth Literacy Questionnaire

The eHLQ has 35 items representing 7 scales that cover the eHLF dimensions: (1) using technology to process health information, (2) understanding of health concepts and language, (3) ability to actively engage with digital services, (4) feel safe and in control, (5) motivated to engage with digital services, (6) access to digital services that work, and (7) digital services that suit individual needs [5,17]. The scale names and construct definitions [17] are shown in Multimedia Appendix 1. Each scale has 4-6 items with 4-point response options: strongly disagree, disagree, agree, and strongly agree, with an assigned value of 1-4, respectively. Scale scores are calculated by averaging the items scores within each scale with equal weighting, generating scale scores that range from 1 to 4.

### Phase 1. Translation and Cultural Adaptation

#### Initial Translation Process

**Figure 1:**
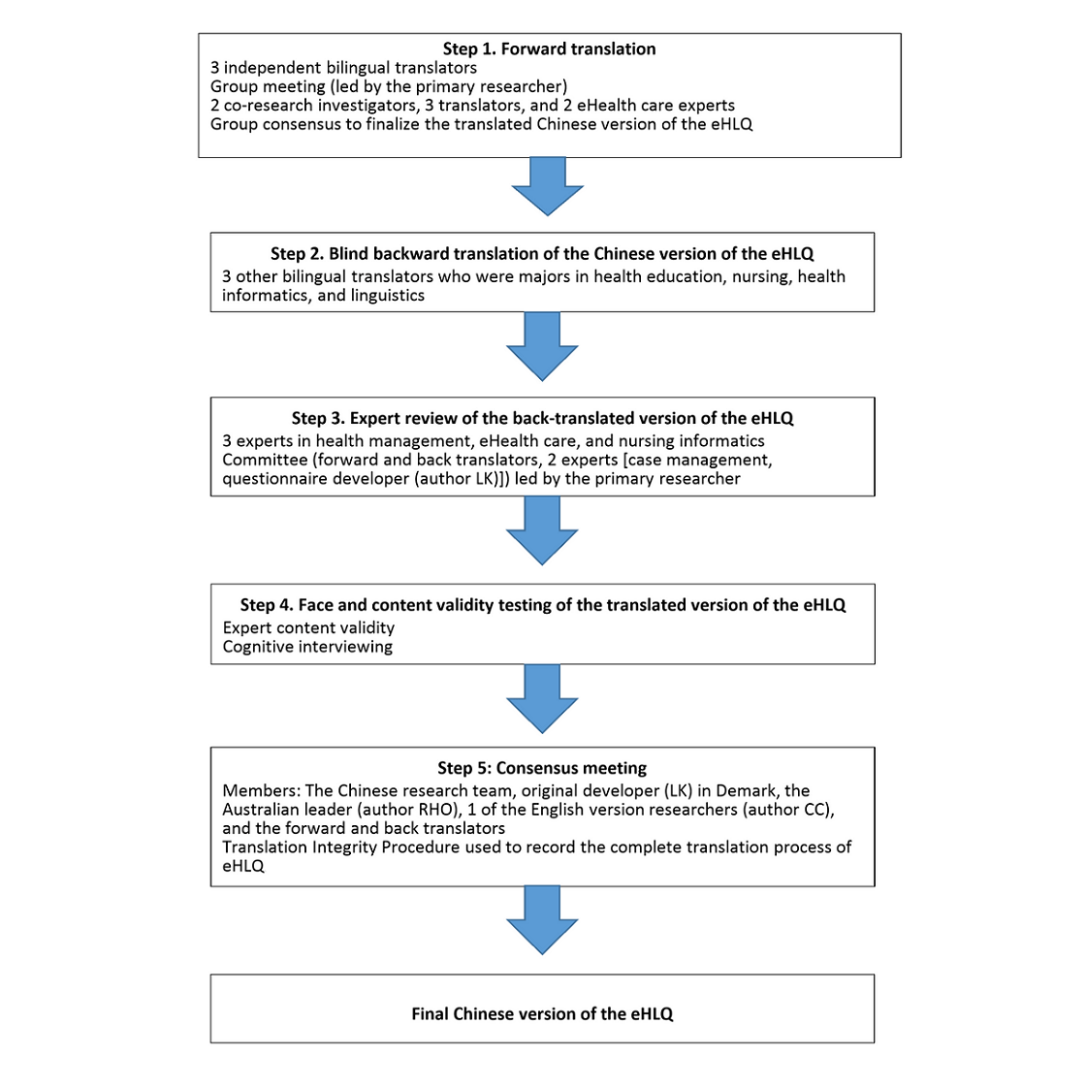
The translation process. eHLQ: eHealth Literacy Questionnaire.

The translation of a questionnaire should not only include a textual change but also consider cultural equivalence and applicability [19-22]. Therefore, this study was designed according to the guidelines for instrument translation, adaptation, and validation proposed by Sousa and Rojjanasrirat [21] and Hall et al [19] and, in particular, the Translation Integrity Procedure developed by Hawkins and Osborne [23] for the eHLQ. The Translation Integrity Procedure ensures that the language translation and cultural adaptation follow detailed item intent descriptions and seek to ensure cultural suitability and measurement equivalence (ie, whether each concept in the translated version is the same strength as that in the original version) [23]. See Figure 1.

Three bilingual translators in both English and Mandarin independently translated the English version of the eHLQ into traditional Chinese, including analysis of cultural appropriateness and measurement equivalence by 5 experts in health care and informatics. Words and phrases that diverged from the original intent were discussed with the translators. Finally, minor modifications of the translated version were incorporated to preserve semantic and idiomatic equivalence in traditional Chinese characters for Mandarin speakers. The translated version was then translated back into English by 3 translators with linguistic qualifications or health or technology qualifications. Two were native English speakers. The forward translation was discussed until a linguistically and culturally equivalent meaning was achieved between the source (guided by item intent and overall construct meaning) and the forward Chinese version.

#### Content Validity and Cognitive Interviewing

Nine experts in nursing, medical practice, public health, informatics, eHealth care, and patient health education were invited to evaluate the content validity of the translated eHLQ. Based on the operational definitions and the item intent of the eHLQ (refer to Kayser et al [17] and the Translation Integrity Procedure [23]), experts evaluated whether the items were representative of the construct and were clearly stated. The level of representativeness was rated on a scale of 1-4, with 4 being representative, 3 being representative but needing minor revision, 2 being representative but needing major revision, and 1 not being representative. Furthermore, the level of clarity was rated on a similar scale, with 4 being clear, 3 being clear but needs minor revision, 2 being clear but needing major revision, and 1 not being clear [24]. The number of experts or participants who assigned 3 points or higher was divided by the total number of people to obtain the content validity index (CVI); a CVI of ≥0.8 indicated that the item had good content validity [24,25].

To check whether people understood the instructions, response format, and items, as intended, cognitive interviews were undertaken [17]. Respondents were asked, “What were you thinking when you were answering that question?” This question was intended to elicit the cognitive process behind the answers. The following prompt was used, if needed: “Why did you select that response option?” Where relevant, items were adjusted, focusing on concepts related to health care and eHealth technologies.

#### Consensus Meeting

The meaning of items in the final translation was verified with the developers (authors LK and RHO) through written reflections on the back translation and a consensus meeting that also included a Mandarin-English bilingual and eHL expert (author CC) and the research team. The purpose was to confirm the forward translation and identify words, phrases, or concepts that were inconsistent with the item intents and implement revisions.

### Phase 2. Psychometric Testing

The Chinese version of the eHLQ was then administered to people with chronic disease to explore its psychometric properties.

#### Recruitment

The participants were a random sample of Chinese adults attending the outpatient departments of the cardiology, nephrology, endocrinology, and family medicine units at several hospitals, such as medical centers, regional hospitals, and district hospitals, in towns or cities in Taiwan. To participate in this study, the inclusion criteria were (1) a diagnosis of type 2 diabetes mellitus, heart disease, or chronic kidney disease for more than 3 months; (2) the ability to clearly communicate in Mandarin and Taiwanese; and (3) age over 20 years. Diabetes, heart disease, and kidney disease were selected because they are the main focus of eHealth care in case management programs in Taiwan. For psychometric testing, a sample size of 300 was considered adequate [21,25].

#### Data Collection Procedures

Participants’ data were collected by trained researchers. Case managers identified potentially eligible participants. At study inception, 1834 eligible people were identified on 2218 lists at 4 health services sites. Each person was randomly assigned a number, and 3 of every 10 (30%) were randomly selected by computer for inclusion in the study. This resulted in 550 (28.99%) people being selected, of which 442 (80.4%) participated. If a participant could not complete the questionnaire due to vision issues, such as presbyopia or myopia, an interviewer assisted the respondent. In the case of any hesitation from respondents during reading, research assistants simply repeated the item verbatim with no additional interpretation. Demographic information was collected, including age, gender, education, employment status, marital status, number of comorbidities, perceived health status in the previous month, income, and activities of daily living. Data were collected from October 2017 to February 2019.

### Ethical Consideration

This study was approved by the institutional review board of the enrolling hospitals (2017-04-002CC; YM104135E2). Written informed consent was obtained prior to data collection.

### Data Analysis

Data analysis was performed using SPSS Statistics version 23 (IBM Corporation, Armonk, NY, USA), STATA version 15.1 (Stata Corporation, College Station, TX, USA), and Mplus Version 8.3 (Muthén & Muthén, Los Angeles, CA, USA) [26]. For demographic characteristics, continuous variables were assessed by means and SD, whereas categorical variables were reported by frequency and percentage. Cronbach α was used to estimate internal consistency, with α≥.7 indicating acceptable reliability [25,27].

To check the assumptions of unidimensionality and local independence for IRT testing, a 1-factor model confirmatory factor analysis (CFA) for each of the 7 scales was fitted to the data using the weighted least squares mean and variance adjusted (WSLMV) estimation available in Mplus. The fit indices comparative fit Index (CFI) and standardized root mean residual (SRMR) were examined based on the 2-index strategy recommended by Shi et al [28] for models with small degrees of freedom. Although the chi-square test is a commonly used fit index, it has been found that it is sensitive to sample size and always rejects models when the sample size is large, while severe deviations from normality may also lead to model rejections [29,30]. The other commonly used fit index root-mean-square error of approximation (RMSEA) was also not an appropriate index for this study as the RMSEA has been found to reject models with small degrees of freedom and using it for assessment can be problematic [28,31]. Indication of a close fit for the CFI was >0.95 and for the SRMR was ≤0.08 [32]. Further investigation of local independence was by inspection of standardized factor loadings, modification indices, and standardized expected parameter change (SEPC) generated in the Mplus output. It has been recommended to use both of these statistics to examine any model misspecification, with a large modification index combined with a positive value of SEPC >0.20 indicating misspecification [33,34].

For IRT analysis, the generalized partial credit model available in STATA version 15.1 was used to estimate item characteristic curves (ICCs), which describe the relationship between a respondent’s ability and how they would respond to an item [35]. The two parameters of the ICC, item discrimination and item location/difficulty, were also evaluated. Item discrimination can detect subtle differences in the respondents’ abilities, and a steeper slope of the ICC indicates a higher discrimination of the item [36,37], while item difficulty shows where the item functions best along the trait scale [35].

Known-group validity was evaluated by exploring associations between eHLQ scales and educational level with one-way ANOVA and Schaffer post hoc testing. *P*<.05 indicated statistical significance. The scales were tested for Gaussian distribution prior to ANOVA. All scales exhibited a normal distribution. Based on a review of previous eHL studies, it would be expected that the educational level would be positively related with all eHLQ scales (ie, lower education would be associated with lower eHLQ scores), although some studies indicate that feeling safe and in control may be inversely related or not related to eHL [38-41]. For the purpose of this study, education was aggregated to 6 International Standard Classification of Education 2011 (ISCED-2011) levels [42]:

- Lower than primary school equivalent to ISCED-2011 levels 0 and 1
- Junior high school equivalent to ISCED-2011 level 2
- Senior high school equivalent to ISCED-2011 level 3
- College equivalent to ISCED-2011 levels 4 and 5
- University equivalent to ISCED-2011 level 6
- Graduate school equivalent to ISCED-2011 levels 7 and 8

## Results

### Demographics and eHealth Literacy Scores

A total of 420 people who met the inclusion criteria completed the questionnaire in full. The response rate was 442 of 550 (80.4%) participants. Reasons for nonparticipation included lack of time, feeling of fatigue, and disinterest. Only 20 of 442 (4.5%) participants were excluded due to missing data.

The participants’ mean (SD) age was 54.7 (13.1) years (range 25-89 years), 280 of 420 (66.7%) were between 50 and 64 years old, the majority (259/420, 61.7%) were male, 136 of 420 (32.4%) had completed junior high school or below, and 219 of 420 (52.1%) were unemployed. The monthly income in the previous year was below New Taiwan dollar (NTD) 20,000 (approximately US $700; the NTD-USD exchange rate was of 2020) for 247 of 420 (58.8%) participants. Regarding health status, most were living with 2 or more chronic diseases (Table 1).

The mean scores of the eHLQ scales ranged from 2.37 to 3.08. Respondents reported the highest scores on scale 2 (understanding of health concepts and language) and the lowest scores on scale 6 (access to digital services that work). See Table 2.

**Table 1:** Characteristics of participants (N=420).

Characteristics			Participants
**Age (years), n (%)**			
	≤50	58 (13.8)	
	51-64	139 (33.1)	
	65-74	141 (33.6)	
	≥75	82 (19.5)	
**Gender, n (%)**			
	Male	259 (61.7)	
	Female	161 (38.3)	
**Education, n (%)**			
	Lower than primary school (ISCED^a^-2011 levels 0 and 1)	82 (19.5)	
	Junior high school (ISCED-2011 level 2)	54 (12.9)	
	Senior high school (ISCED-2011 level 3)	69 (16.4)	
	College (ISCED-2011 levels 4 and 5)	117 (27.9)	
	University (ISCED-2011 level 6)	65 (15.5)	
	Graduate school (ISCED-2011 levels 7 and 8)	33 (7.9)	
**Employment status, n (%)**			
	Not working (retired or unemployed)	219 (52.1)	
	Working	201(47.9)	
**Marital status, n (%)**			
	Single	147 (35.0)	
	Married or with a partner	273 (65.0)	
**Chronic disease, n (%)^b^**			
	Diabetes mellitus	96 (22.9)	
	Hypertension	249 (59.3)	
	Cardiovascular disease	261 (62.1)	
	Hyperlipidemia	104 (24.8)	
	Chronic kidney disease	175 (41.7)	
**Health status, n (%)**			
	1 disease	53 (12.7)	
	2 diseases	198 (47.1)	
	≥3 diseases	169 (40.2)	
**Monthly income in the last year, n (%)**			
	<NTD^c^ 20,000 (~US $700) low	247 (58.8)	
	NTD 20,000-40,000 (~US $700-1400) lower middle	74 (17.6)	
	NTD 40,001-60,000 (~US $700-1800) middle	43 (10.2)	
	>NTD 60,000 (~>US $1800) middle higher	56 (13.3)	
**Living status, n (%)**			
	Lived alone	47 (11.2)	
	With spouse	227 (54.1)	
	With children	146 (34.8)	

^a^ISCED: International Standard Classification of Education.

^b^More than 1 response was possible.

^c^NTD: New Taiwan dollar.

**Table 2:** The eHealth Literacy Questionnaire (eHLQ) scale scores and internal consistency.

Scale number	Scale name	Mean (SD)	Cronbach α
1	Using technology to process health information	2.41 (0.95)	.95
2	Understanding of health concepts and language	3.08 (0.57)	.75
3	Ability to actively engage with digital services	2.45 (0.91)	.90
4	Feel safe and in control	2.73 (0.78)	.87
5	Motivated to engage with digital services	2.49 (0.96)	.93
6	Access to digital services that work	2.37 (0.84)	.91
7	Digital services that suit individual needs	2.45 (0.99)	.90

### Phase 1. Translation

Ten experts reviewed the item content validity, which resulted in scale CVIs from 0.88 to 0.95, well above the acceptable level of 0.80. For the item-level CVI, the lowest score was 0.80 (items 3, 17, 18, and 34), which was acceptable.

A total of 45 people participated in a cognitive interview. Most interviewees suggested some words or terms that required more description for clarity and ease of understanding. For instance, they were unsure of terms covering health technology services, people who required health information, and authorized people. In addition, they did not easily link these terms to their disease management situation and the relevant health care system. The instructions and definitions of terms in the questionnaire introduction were therefore revised. All other items were understood as intended and no further changes made. The interview process took 10-15 minutes to complete.

The consensus meeting identified 4 items that required minor refinement (items 6, 19, 24, and 25). For example, for items 19 and 24, the word “find” was originally translated as “發現.” However, the consensus meeting revealed that “find” includes the meaning “perceive” and “believe” and so was replaced with “發覺” to reflect the intended meaning. See Multimedia Appendix 2 for the final Chinese version of the eHLQ.

### Phase 2. Psychometric Testing

#### Reliability

The internal consistency coefficients are shown in Table 2. All scales had α>.80 except scale 2, which had α=.75.

#### Known-Group Validity

There were striking differences among the educational levels, with a clear monotonic increase in scores for all scales from the lowest to the highest education, except for the higher levels of education for scale 2. A comparison of the 7 scales of the Chinses version of the eHLQ across educational levels is shown in Multimedia Appendix 3. The largest differences between the lowest and highest education levels were for scale 6, where people with the lowest education, on average, scored 1.08, indicating that almost all respondents strongly disagreed that they could access technologies that worked. The smallest differences in education were seen for scale 2, where the average score of the lowest-education group was 2.89 compared with 3.24 in the highest-education group.

#### Construct Validity

The 1-factor CFA models generally fitted the data well on all scales based on the CFI and SRMR fit indices, and the SEPC values were below 0.2 except for scale 2, with CFI=0.95, SRMR=0.04, the largest modification index=86.2 for eHLQ26 (“I use measurements about my body to help me understand my health”) and eHLQ15 (“I understand medical results about me”), and SEPC=0.29. This finding indicates that content within these 2 particular items is related in a unique way, in addition to how they are related to the latent variable of the scale’s construct. A model with a correlated residual between these 2 items was tested, and the results demonstrated a close fit, with no large modification index or SEPC (see Multimedia Appendix 4). Standardized factor loadings were significant for all scales, with loadings >.50 (Multimedia Appendix 5). As such, the unidimensionality and local independence assumptions were met, except for scale 2, for which the local independence assumption might not hold and the IRT results for this scale needed to be interpreted with caution.

#### Item Response Theory

IRT analysis showed that respondents could use the response options in a consistent way and that no items were found to have disordered thresholds. See Multimedia Appendix 6 for the ICCs of the Chinese version of the eHLQ. Inspection of the steepness of the slopes of the ICCs and the estimated item discrimination parameters showed that all items except items 15 and 26 of scale 2 had acceptable-to-good discrimination between people with different levels of ability. The estimated item difficulty parameters demonstrated a range of difficulty levels within each scale except scale 7 (digital services that suit individual needs). The widest difficulty range was noted for scale 4 (feel safe and in control; range 0.82-1.59) and scale 6 (range 0.82-1.59). Scale 7 had the narrowest range (0.70-0.79). However, all results within the scales were statistically significantly different (Table 3).

**Table 3:** Item response theory (IRT) analysis of the Chinese version of the eHealth Literacy Questionnaire (eHLQ) using the generalized partial credit model.

Scale item		Item difficulty (95% CI)	Item discrimination (95% CI)
**1. Using technology to process health information**			
	eHLQ7	0.69 (0.57-0.81)	7.48 (6.08-8.89)
	eHLQ11	0.84 (0.70-0.97)	5.73 (4.67-6.79)
	eHLQ13	0.76 (0.64-0.89)	6.53 (5.34-7.72)
	eHLQ20	0.89 (0.73-1.02)	5.21 (4.24-6.19)
	eHLQ25	1.02 (0.85-1.18)	4.30 (3.50-5.09)
**2. Understanding of health concepts and language**			
	eHLQ5	0.92 (0.67-1.17)	1.18 (0.93-1.44)
	eHLQ12	1.19 (0.89-1.48)	1.08 (0.85-1.30)
	eHLQ15	0.83 (0.22-1.44)	0.38 (0.22-0.53)
	eHLQ21	1.40 (1.01-1.80)	0.78 (0.60-0.96)
	eHLQ26	1.05 (0.13-1.97)	0.27 (0.11-0.44)
**3. Ability to actively engage with digital services**			
	eHLQ4	0.82 (0.69-0.96)	5.10 (4.16-6.04)
	eHLQ6	0.76 (0.66-0.92)	6.06 (4.96-7.17)
	eHLQ8	0.79 (0.66-0.94)	4.28 (3.49-5.08)
	eHLQ17	1.09 (0.76-1.34)	5.32 (4.34-6.30)
	eHLQ32	0.84 (0.70-0.97)	5.89 (4.80-6.97)
**4.** **Feel safe and in control**			
	eHLQ1	0.82 (0.56-1.09)	1.05 (0.83-1.27)
	eHLQ10	1.59 (1.05-1.80)	0.98 (0.77-1.19)
	eHLQ14	1.25 (0.95-1.54)	1.24 (0.99-1.49)
	eHLQ22	0.93 (0.67-1.19)	1.12 (0.89-1.35)
	eHLQ30	1.13 (0.84-1.42)	1.09 (0.87-1.32)
**5.** **Motivated to engage with digital services**			
	eHLQ2	0.68 (0.64-0.91)	4.41 (3.59-5.23)
	eHLQ19	0.59 (0.51-0.80)	9.55 (7.73-11.36)
	eHLQ24	0.85 (0.70-0.96)	7.30 (5.93-8.68)
	eHLQ27	0.80 (0.68-0.95)	5.83 (4.75-6.92)
	eHLQ35	0.77 (0.64-0.90)	5.11 (4.18-6.05)
**6. Access to digital services that work**			
	eHLQ3	1.67 (1.33-2.02)	1.26 (1.01-1.51)
	eHLQ9	0.83 (0.70-0.97)	5.38 (4.39-6.37)
	eHLQ16	1.10 (0.90-1.33)	2.22 (1.81-2.63)
	eHLQ23	0 .90 (0.75-1.05)	4.17 (3.40-4.94)
	eHLQ29	1.12 (0.91-1.32)	2.56 (2.08 -3.03)
	eHLQ34	0.89 (0.75-1.05)	4.44 (3.60-5.28)
**7. Digital services that suit individual needs**			
	eHLQ18	0.71 (0.65-0.89)	12.15 (9.58-14.73)
	eHLQ28	0.77 (0.65-0.89)	9.60 (7.72-11.47)
	eHLQ31	0.79 (0.67-0.91)	10.51 (8.39-12.63)
	eHLQ33	0.73 (0.61-0.85)	10.63 (8.52-12.73)

## Discussion

### Principal Findings

This study undertook a rigorous process of translating the eHLQ, ensuring cultural appropriateness for the Chinese context, and examined several key indicators of validity based on data derived from a large randomly selected sample of people with chronic conditions from diverse demographic backgrounds. In this setting, the eHLQ was found to have strong-to-acceptable psychometric properties using both classical test and IRT approaches. The translated and culturally adapted eHLQ items were found to be highly coherent with the original intended meanings and psychometric properties.

For a translated version to be considered a robust questionnaire in this setting, not only are systematic and standardized translation processes required but also a verification process [16,20,21,43,44]. Our translation process provides evidence for a validity argument of the translated version, as recommended by Hawkins et al [19,20,43]. The Translation Integrity Procedure with the detailed construct and item intent descriptions supported this process with a common foundation for the translation team to negotiate the nuances of item meanings to maximize construct equivalence, minimize threats to construct validity during the translation process, and generate qualitative validity evidence for score interpretation and use in a new linguistic context [20]. Overall, the process ensured that the translated version had semantic, idiomatic, experiential, and conceptual equivalence with the original [21,45].

All of the items within the scales loaded strongly on their respective factors. With 1 modification, all 1-factor models fitted the data well. For scale 2, a correlated residual was added between 2 items, which may have been independently related due to the hospital setting, where the content of both items related to medical results and measurement about one’s body (items 15 and 26, respectively). Cronbach α, which is frequently inflated due to excess items within a scale, was >.85 for all scales except Scale 2, which still had an acceptable reliability of .75. Consistent with the validity-driven approach, the development of the eHLQ ensured that a minimal number of items (4-6 for the eHLQ scales) were carefully generated to capture the full breadth of each individual construct. This ambitious parsimonious constraint was reproduced in the Mandarin language setting, which included people with diverse educational levels and health conditions. The original Danish validity-testing study reported similar internal consistency (range .77-.86) [17], where scale 2 also had the lowest value. Of note, the internal consistency of 3 scales (1, 5, and 6) was greater than .90, which may indicate some translated items may be understood in an overly similar way in this Chinese population [46]. In addition, almost 60% of participants used case management services and had the same experience in eHealth care in using mobile health monitors, such as blood sugar and blood pressure, so it is possible that most respondents had similar experiences relative to multiple items within scales. Importantly, for all the scales except scale 7, a range of difficulties was found, indicating that differences among individuals were expressed across items.

Our study showed that the average scores of people with primary or lower education is substantially lower than those of other more educated groups. The findings were striking, especially for scale 6, where the average score was 1.08, which indicates that almost all respondents with primary or lower education responded “strongly disagree” on all items in this scale. In contrast, for the highest-education group, most respondents indicated they “agreed”, with a score of 2.93, indicating they had access to digital services that work. Similar patterns were also seen for scales 1, 3, 5, and 7; however, the lowest-education group, on average, on all items per scale marked “disagree” rather than “strongly disagree.” These findings demonstrate a stark social gradient related to education, where people with higher education clearly have higher eHL than those with lower education. This is generally in line with other findings that lower education is associated with lower eHL [9,38-40]. In a study of nursing students in Denmark, which also used the eHLQ, found that graduate-level students scored higher than entry-level nursing students on scales 1-3, with no differences on the other scales [47]. The eHLQ appears to be a promising tool to understand digital access to different educational categories and is therefore likely to be a useful tool for understanding socioeconomic determinants of digital access inequity.

IRT analysis also provided insights into the psychometric properties of the eHLQ. This analysis showed good sensitivity in detecting participants with different levels of ability, as well as representing a range of difficulty within each scale, echoing the findings of the initial development studies in Denmark [17]. Only 2 items relating to medical results and measurements, items 15 and 26 of scale 2, were found to have low item discrimination. These items were also found to be problematic in classical test theory analysis (reliability and CFA, as noted in the Construct Validity section). In this health care setting, respondents may be prone to providing socially desirable answers, common in Chinese culture. People tend to be cautious to ask and share problems with health care professionals to avoid discrimination [48,49], or they may tend to hide a lack of understanding about their tests or measurements. Although it is possible that the performance of these items relates to the particular characteristics of the respondents in our study, further work in different populations alongside linguistic evaluation will shed light on these items.

### Strengths

An important strength of this study was the heterogeneity of the sample. People with a range of chronic diseases from various hospitals and clinics were included. These are key settings and populations for the application of the eHLQ in future studies, and given that overall good psychometric properties were observed in this diverse sample, it is likely to be a robust measure in other related settings.

### Limitations

The CFA results indicated that the local independence assumption for scale 2 might not have been met; therefore, the IRT results for this scale need to be interpreted with caution. This study relied on self-report, and therefore, the respondents’ answers may be prone to recall bias and social desirability, similar to other self-report measures. Some participants completed the questionnaire with the help of a research assistant. This only occurred on 42 occasions, and although the administration mode may affect the respondents’ scores, this was regarded as an important process to ensure people with low eHL were included to maximize the representativeness of the sample. Future research should explore whether the administration mode introduces bias.

### Conclusion

This study demonstrates that the eHLQ has good linguistic equivalence and psychometric properties, following a rigorous translation and cultural adaptation process and extensive psychometric testing using both classical test theory and IRT approaches. The 7 scales of the eHLQ can efficiently assess diverse dimensions of eHL of people across chronic diseases. The questionnaire is likely to enable health care providers and eHealth system developers to better understand people’s ability to engage with and use technology so that these systems can be developed, evaluated, and redesigned to meet the health and equity needs of their communities. As such, the eHLQ can be used as a reference to design adaptive care programs to improve the quality and effectiveness of care. This may also help avoid the health disparities created by the advancement of digital technologies.

## Appendix

Multimedia Appendix 1 Scale names and construct definitions of the eHealth Literacy Questionnaire (eHLQ).

Multimedia Appendix 2 Scales of the Chinese version of the eHealth Literacy Questionnaire (eHLQ).

Multimedia Appendix 3 Comparison of the 7 scales of the Chinese version of the eHealth Literacy Questionnaire (eHLQ) across educational levels.

Multimedia Appendix 4 Model fit indices for the 1-factor confirmatory factor analysis of the Chinese version of the eHealth Literacy Questionnaire (eHLQ).

Multimedia Appendix 5 Standardized factor loadings of the 7 one-factor models of the Chinese version of the eHealth Literacy Questionnaire (eHLQ).

Multimedia Appendix 6 Item characteristic curves (ICCs) of the Chinese version of the eHealth Literacy Questionnaire (eHLQ).

## Abbreviations

CFA: confirmatory factor analysis
CFI: comparative fit index
CVI: content validity index
eHealth: electronic health
eHL: eHealth literacy
eHLF: eHealth Literacy Framework
eHLQ: eHealth Literacy Questionnaire
ICC: item characteristic curve
IRT: item response theory
ISCED: International Standard Classification of Education
RMSEA: root-mean-square error of approximation
SEPC: standardized expected parameter change
SRMR: standardized root mean residual
