# Supplementary material for: Validity Testing and Cultural Adaptation of the eHealth Literacy Questionnaire (eHLQ) Among People With Chronic Diseases in Taiwan: Mixed Methods Study
Source: J Med Internet Res. 2022 Jan 19;24(1):e32855. doi: 10.2196/32855 (PMC8811686; doi:10.2196/32855)
Supplement: Multimedia Appendix 1 [file jmir_v24i1e32855_app1.docx]

Multimedia Appendix 1. The scale names and construct definitions of eHealth Literacy Questionnaire (eHLQ)**^17^**

| **No.** | **Scale name** | **Scale definition** |
| --- | --- | --- |
| 1 | Using technology to process health information  (5 items) | Able to read, write, and remember, apply basic numerical concepts, and understand context-specific language (e.g. health, technology or English) as well as critically appraise information. Know when, how and what information to use. |
| 2 | Understanding of health concepts and language  (5 items) | Know about basic physiological functions and own current health status. Aware of risk factors and how to avoid them or reduce their influence on own health as well as navigating the health care system. |
| 3 | Ability to actively engage with digital services  (5 items) | Being comfortable using digital services for handling information. |
| 4 | Feel safe and in control  (5 items) | Feel that they have the ownership of personal data stored in the systems and that the data are safe and can be accessed only by people to whom they are relevant (such as own doctor and nurse). |
| 5 | Motivated to engage with digital services  (5 items) | Feel that engaging in the use of digital services will be useful for them in managing their health. |
| 6 | Access to digital services that work  (6 items) | Have access to digital services that the users trust to be working when they need it and as they expect it to work. |
| 7 | Digital services that suit individual needs  (4 items) | Have access to digital services that suit the specific needs and preferences of the users. This includes responsive features of both the information technology and health care system as well as adaptation of devices and interfaces to be used by people with physical and mental disabilities. |
