# Supplementary material for: Validity Testing and Cultural Adaptation of the eHealth Literacy Questionnaire (eHLQ) Among People With Chronic Diseases in Taiwan: Mixed Methods Study
Source: J Med Internet Res. 2022 Jan 19;24(1):e32855. doi: 10.2196/32855 (PMC8811686; doi:10.2196/32855)
Supplement: Multimedia Appendix 2 [file jmir_v24i1e32855_app2.docx]

Multimedia Appendix 2. The scales of the Chinese version of eHealth Literacy Questionnaire(eHLQ)

| Chinese version of eHealth Literacy Questionnaire scale names and items^a^ | |
| --- | --- |
| 1.運用科技來處理健康資訊 | |
|  | 我使用科技找…… |
|  | 我經常使用科技…… |
|  | 科技幫助我決定怎樣…… |
|  | 我使用科技分享……. |
|  | 我使用科技來…… |
| 2.了解健康概念和語言 | |
|  | 我具備的知識有助於…… |
|  | 關於我的健康我有足夠….. |
|  | 我了解關於我自己……. |
|  | 整體而言，我了解我的…… |
|  | 我使用身體的測量….. |
| 3.主動參與數位服務的能力 | |
|  | 我知道如何使用科技取得…….. |
|  | 我知道如何受惠於科技…… |
|  | 我能夠將資料輸入….. |
|  | 我很快地學會如何….. |
|  | 我很容易學會….. |
| 4.感到安全和控制 | |
|  | 我確定只有應該使用的人……. |
|  | 我的電子健康照護…… |
|  | 我清楚地了解健康照護人員…… |
|  | 我確定只有被授權的人…… |
|  | 我有信心健康照護人員….. |
| 5.被激發參與數位服務 | |
|  | 科技讓我覺得能主動……. |
|  | 我發覺科技可幫助…. |
|  | 當我使用科技時….. |
|  | 科技促進我….. |
|  | 我發覺科技有助…… |
| 6.取得的數位服務是可用的 | |
|  | 需要關於我健康資訊的人…… |
|  | 我的健康照護人員提供的服務…….. |
|  | 不論我在任何地方…… |
|  | 所有我使用的健康科技…… |
|  | 我的大部分醫療照護人員…… |
|  | 我接觸過的健康科技…… |
| 7.數位服務符合個人需求 | |
|  | 我發覺健康科技服務….. |
|  | 我發覺健康科技….. |
|  | 我發覺健康科技服務…… |
|  | 健康科技服務提供…… |

^a^Items are truncated. The full list of items is available with the authors.
