# Supplementary material for: Validity Testing and Cultural Adaptation of the eHealth Literacy Questionnaire (eHLQ) Among People With Chronic Diseases in Taiwan: Mixed Methods Study
Source: J Med Internet Res. 2022 Jan 19;24(1):e32855. doi: 10.2196/32855 (PMC8811686; doi:10.2196/32855)
Supplement: Multimedia Appendix 3 [file jmir_v24i1e32855_app3.docx]

Multimedia Appendix 3**.** Comparison of the 7 scales of the Chinese version of eHealth Literacy Questionnaire(eHLQ) across educational levels (n=420)

| Variable | 1  Using technology to process health information | | | 2  Understanding of health concepts and language | | | 3  Ability to actively engage with digital services | | |
| --- | --- | --- | --- | --- | --- | --- | --- | --- | --- |
|  | Mean(SD)^a^ | ANOVA | *P value* | Mean(SD) | ANOVA | *P value* | Mean(SD) | ANOVA | *P value* |
| Education |  | 15.3 | <.01 |  | 4.9 | .004 |  | 14.7 | <.01 |
| ≦Primary school① | 1.75(0.82) | ①<②③  <④⑤⑥ |  | 2.89(0.56) |  |  | 1.79(0.93) | ①<  ③④⑤⑥ |  |
| Junior high school② | 2.15(0.80) | ②<⑤<⑥ |  | 2.94(0.51) |  |  | 2.24(0.80) | ②<⑤⑥ |  |
| Senior high school③ | 2.47(0.87) | ③>①  ③<⑥ |  | 3.02(0.52) |  |  | 2.46(0.72) | ③>①  ③<⑥ |  |
| College④ | 2.51(0.98) | ④>②>①  ④<⑥ |  | 3.24(0.54) |  |  | 2.59(0.98) | ④>① |  |
| University⑤ | 2.64(0.90) | ⑤>②>① |  | 3.07(0.65) |  |  | 2.66(0.87) | ⑤>②>① |  |
| Graduate school⑥ | 3.12(0.66) | ⑥>①②③④ |  | 3.24(0.52) |  |  | 3.13(0.65) | ⑥>  ③②① |  |

^a^ SD=standard deviation

Multimedia Appendix 3. Comparison of the 7 scales of the Chinese version of eHealth Literacy Questionnaire(eHLQ) across educational levels (n=420) (cont.)

| Variable | 4  Feel safe and in control | |  | 5  Motivated to engage with digital services | |  |
| --- | --- | --- | --- | --- | --- | --- |
|  | Mean(SD)^a^ | ANOVA | *P value* | Mean(SD) | ANOVA | *P value* |
| Education |  | 7.8 | <.01 |  | 16.1 | <.01 |
| ≦ Primary school① | 2.34(0.83) | ①<  ③④⑤⑥ |  | 1.77(0.81) | ①<③  <④⑤⑥ |  |
| Junior high school② | 2.51(0.72) | ②<⑤⑥ |  | 2.28(0.84) | ②<⑤⑥ |  |
| Senior high school③ | 2.78(0.73) | ③>① |  | 2.53(0.87) | ③>①  ③<⑥ |  |
| College④ | 2.81(0.78) | ④>① |  | 2.67(0.99) | ④>① |  |
| University⑤ | 2.94(0.68) | ⑤>① |  | 2.71(0.92) | ⑤>②① |  |
| Graduate school⑥ | 3.07(0.66) | ⑥>①② |  | 3.18(0.63) | ⑥>  ③②① |  |

^a^ SD=standard deviation

Multimedia Appendix 3. Comparison of the 7 scales of the Chinese version of eHealth Literacy Questionnaire (eHLQ) across educational levels (n=420) (cont.)

| Variable | 6  Access to digital services that work | |  | 7  Digital services that suit individual needs | |  |
| --- | --- | --- | --- | --- | --- | --- |
|  | Mean(SD)^a^ | ANOVA | *P value* | Mean(SD) | ANOVA | *P value* |
| Education |  | 12.2** | <.01 |  | 14.4** | <.01 |
| ≦ Primary school① | 1.08(0.70) | ①<  ③④⑤⑥ |  | 1.77(0.86) | ①<  ③④⑤⑥ |  |
| Junior high school② | 2.27(0.71) | ②<⑥ |  | 2.18(0.83) | ②<⑤⑥ |  |
| Senior high school③ | 2.44(0.78) | ③>① |  | 2.51(0.91) | ③>①  ③<⑥ |  |
| College④ | 2.48(0.90) | ④>① |  | 2.61(1.04) | ④>① |  |
| University⑤ | 2.50(0.83) | ⑤>① |  | 2.66(0.93) | ⑤>②① |  |
| Graduate school⑥ | 2.93(0.59) | ⑥>②① |  | 3.15(0.70) | ⑥>  ③②① |  |

^a^ SD=standard deviation
