# Supplementary material for: Validity Testing and Cultural Adaptation of the eHealth Literacy Questionnaire (eHLQ) Among People With Chronic Diseases in Taiwan: Mixed Methods Study
Source: J Med Internet Res. 2022 Jan 19;24(1):e32855. doi: 10.2196/32855 (PMC8811686; doi:10.2196/32855)
Supplement: Multimedia Appendix 4 [file jmir_v24i1e32855_app4.docx]

Multimedia Appendix 4. Model fit indices for the one-factor confirmatory factor analysis of the Chinese version of eHealth Literacy Questionnaire(eHLQ)

| **Scale** | **CFI^a^** | **SRMR^b^** | **Largest M.I.^c^** | **Std E.P.C.^d^** |
| --- | --- | --- | --- | --- |
| 1. Using technology to process health information | 1.00 | 0.01 | 66.04  (eHL11 with eHLQ7) | 0.06 |
| 1. Understanding of health concepts and language^e^ | 1.00 | 0.01 | 6.4  (eHLQ15 with eHLQ12) | -0.08 |
| 1. Ability to actively engage with digital services | 0.99 | 0.02 | 101.52  (eHLQ32 with eHLQ17) | 0.12 |
| 1. Feel safe and in control | 0.99 | 0.02 | 26.34  (eHLQ22 with eHLQ14) | -0.11 |
| 1. Motivated to engage with digital services | 1.00 | 0.01 | 50.39  (eHLQ24 with eHLQ2) | -0.01 |
| 1. Access to digital services that work | 0.99 | 0.02 | 70.82  (eHLQ34 with eHLQ23) | 0.10 |
| 1. Digital services that suit individual needs | 1.00 | 0.01 | 41.39  (eHLQ33 with eHLQ18) | -0.04 |

^a^CFI = comparative fit index

^b^SRMR = standardized root mean residual

^c^M.I. = modification indices

^d^Std E.P.C. = Standardized expected parameter change

^e^Scale 2 model with added correlated residual
