# Supplementary material for: Validity Testing and Cultural Adaptation of the eHealth Literacy Questionnaire (eHLQ) Among People With Chronic Diseases in Taiwan: Mixed Methods Study
Source: J Med Internet Res. 2022 Jan 19;24(1):e32855. doi: 10.2196/32855 (PMC8811686; doi:10.2196/32855)
Supplement: Multimedia Appendix 5 [file jmir_v24i1e32855_app5.docx]

Multimedia Appendix 5. Standardized factor loadings of the seven one-factor models of the Chinese version of eHealth Literacy Questionnaire(eHLQ)

| **Scale** | **Item** | **Factor loadings^a^ (95% CI)** |
| --- | --- | --- |
| 1. Using technology to process health information | eHLQ7 | 0.99 (0.98 – 1.00) |
|  | eHLQ11 | 0.96 (0.95 – 0.97) |
|  | eHLQ13 | 0.95 (0.94 – 0.96) |
|  | eHLQ20 | 0.91 (0.89 – 0.92) |
|  | eHLQ25 | 0.85 (0.83 – 0.88) |
| 1. Understanding of health concepts and language | eHLQ5 | 0.80 (0.76 – 0.84) |
|  | eHLQ12 | 0.80 (0.76 – 0.84) |
|  | eHLQ15 | 0.58 (0.51 – 0.65) |
|  | eHLQ21 | 0.72 (0.67 – 0.77) |
|  | eHLQ26 | 0.56 (0.50 – 0.63) |
| 1. Ability to actively engage with digital services | eHLQ4 | 0.90 (0.88 – 0.92) |
|  | eHLQ6 | 0.91 (0.89 – 0.93) |
|  | eHLQ8 | 0.87 (0.84 – 0.90) |
|  | eHLQ17 | 0.93 (0.91 – 0.95) |
|  | eHLQ32 | 0.92 (0.90 – 0.94) |
| 1. Feel safe and in control | eHLQ1 | 0.82 (0.79 – 0.86) |
|  | eHLQ10 | 0.82 (0.79 – 0.86) |
|  | eHLQ14 | 0.78 (0.74 – 0.82) |
|  | eHLQ22 | 0.85 (0.82 – 0.89) |
|  | eHLQ30 | 0.78 (0.74 – 0.82) |
| 1. Motivated to engage with digital services | eHLQ2 | 0.89 (0.87 – 0.91) |
|  | eHLQ19 | 0.99 (0.98 – 0.99) |
|  | eHLQ24 | 0.97 (0.97 – 0.98) |
|  | eHLQ27 | 0.92 (0.91 – 0.94) |
|  | eHLQ35 | 0.93 (0.91 – 0.94) |
| 1. Access to digital services that work | eHLQ3 | 0.69 (0.64 – 0.74) |
|  | eHLQ9 | 0.91 (0.89 – 0.93) |
|  | eHLQ16 | 0.84 (0.81 – 0.87) |
|  | eHLQ23 | 0.93 (0.91 – 0.94) |
|  | eHLQ29 | 0.87 (0.85 – 0.90) |
|  | eHLQ34 | 0.90 (0.88 – 0.92) |
| 1. Digital services that suit individual needs | eHLQ18 | 0.97 (0.96 – 0.98) |
|  | eHLQ28 | 0.96 (0.96 – 0.98) |
|  | eHLQ31 | 0.98 (0.97 – 0.99) |
|  | eHLQ33 | 0.98 (0.97 – 0.98) |

Factor loadings^a^ **:** All loadings are significant at p < .01
