# Supplementary material for: Validity Testing and Cultural Adaptation of the eHealth Literacy Questionnaire (eHLQ) Among People With Chronic Diseases in Taiwan: Mixed Methods Study
Source: J Med Internet Res. 2022 Jan 19;24(1):e32855. doi: 10.2196/32855 (PMC8811686; doi:10.2196/32855)
Supplement: Multimedia Appendix 6 [file jmir_v24i1e32855_app6.docx]

**Multimedia Appendix 6.** Item characteristic curves (ICCs) of the Chinese version of eHealth Literacy Questionnaire(eHLQ)

Scale1.Using technology to process health information

Scale 2. Understanding of health concepts and language

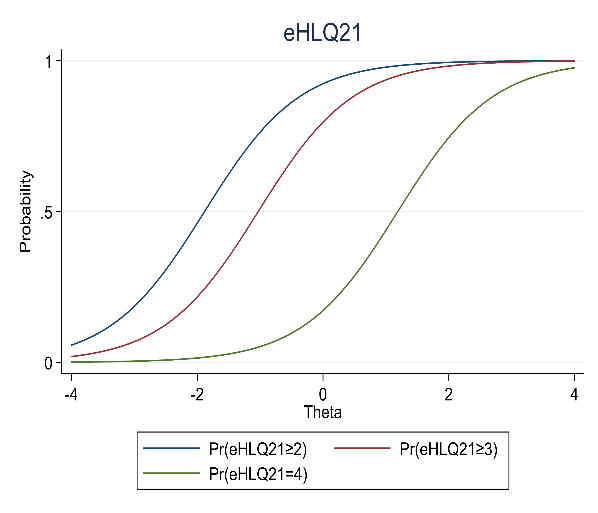


Scale 3. Ability to actively engage with digital services

Scale 4. Feel safe and in control

Scale 5. Motivated to engage with digital services

Scale 6. Access to digital services that work

Scale 7. Digital services that suit individual needs.
